# Novel Pyrimidine Derivatives as Potential Anticancer Agents: Synthesis, Biological Evaluation and Molecular Docking Study

**DOI:** 10.3390/ijms22083825

**Published:** 2021-04-07

**Authors:** Beata Tylińska, Benita Wiatrak, Żaneta Czyżnikowska, Aneta Cieśla-Niechwiadowicz, Elżbieta Gębarowska, Anna Janicka-Kłos

**Affiliations:** 1 Department of Organic Chemistry, Wroclaw Medical University, Borowska 211A, 50-556 Wroclaw, Poland; 2 Department of Pharmacology, Wroclaw Medical University, Mikulicza-Radeckiego 2, 50-345 Wrocław, Poland; 3 Department of Inorganic Chemistry, Wroclaw Medical University, Borowska 211A, Borowska 211A, 50-556 Wroclaw, Poland; zaneta.czyznikowska@umed.wroc.pl (Ż.C.); anna.janicka-klos@umed.wroc.pl (A.J.-K.); 4 Department of Basic Medical Sciences, Wroclaw Medical University, Borowska 211A, 50-556 Wroclaw, Poland; aneta.ciesla-niechwiadowicz@umed.wroc.pl; 5 Agricultural Microbiology Lab, Department of Plant Protection, Wrocław University of Environmental and Life Sciences, Grunwaldzka 53, 50-375 Wrocław, Poland; elzbieta.gebarowska@upwr.edu.pl

**Keywords:** pyrimidine, anticancer, 3,4-dihydronaphthalen, 6-hydrazinopyrimidine, lipophilicity, QSAR study, topoisomerase II, DNA intercalating

## Abstract

In the present paper, new pyrimidine derivatives were designed, synthesized and analyzed in terms of their anticancer properties. The tested compounds were evaluated in vitro for their antitumor activity. The cytotoxic effect on normal human dermal fibroblasts (NHDF) was also determined. According to the results, all the tested compounds exhibited inhibitory activity on the proliferation of all lines of cancer cells (colon adenocarcinoma (LoVo), resistant colon adenocarcinoma (LoVo/DX), breast cancer (MCF-7), lung cancer (A549), cervical cancer (HeLa), human leukemic lymphoblasts (CCRF-CEM) and human monocytic (THP-1)). In particular, their feature stronger influence on the activity of P-glycoprotein of cell cultures resistant to doxorubicin than doxorubicin. Tested compounds have more lipophilic character than doxorubicin, which determines their affinity for the molecular target and passive transport through biological membranes. Moreover, the inhibitory potential against topoisomerase II and DNA intercalating properties of synthesized compounds were analyzed via molecular docking.

## 1. Introduction

As reported by the WHO, 18 million people are presently living with cancer; over 9 million people died from cancer in 2018. Due to the lack of effective and selectively acting anticancer therapies, these numbers are still increasing. The compounds based on the scaffold of pyrimidine exhibit a broad spectrum of pharmacological activity such as anti-inflammatory [1], antimicrobial [2,3], anti-HIV [2], antidiabetic [4] and anticancer activity [5,6,7]. The most recognized drugs based on analogs of pyrimidines are antibacterial (sulfadiazine, trimethoprim), antiviral (trifluridine, idoxuridine), anti-malarial (sulfadoxine), anti-HIV (Retrovir (zidovudine), stavudine), anti-tuberculosis (viomycin), anticancer (5-fluorouracil) agents. The data also show that the adjunction of a [(dialkylamino)alkyl]amino or (hydroxyalkyl)amino side chain enhances anticancer activity of compounds [8,9,10,11]. There are also studies on novel derivatives with a 3,4-dihydronaphthalen moiety that exhibit anticancer activity [12,13]. Another important chemical group that is present in biologically active agents is hydrazone. It exhibits a broad spectrum of anticancer and antimicrobial activity [14,15,16,17,18,19]. The data obtained from the structure–activity relationships (SAR) analysis indicate that combined structures of pyrimidine, 3,4-dihydronaphthalene and alkylamine might have a synergistic anticancer effect. 

In the present paper, we analyzed and described the anticancer activity of newly designed compounds, a hybrid of pyrimidine–hydrazone moieties, dihydronaphthalene and alkylamine chain. All pyrimidine derivatives were evaluated in vitro for their antitumor activity against LoVo colon adenocarcinoma, LoVo/DX-resistant colon adenocarcinoma, MCF-7 breast cancer, A549 lung cancer, cervical cancer (HeLa), human leukemic lymphoblasts (CCRF-CEM) and human monocytic (THP-1) cell lines. We also evaluated their chemical and physical properties to determine the ability to be an orally active drug in humans. Basically, several different modes of action of the anticancer drug are recognized.

One of the possibilities is controlling transcription factors and polymerases where drugs interact directly with the protein bound to DNA. Another is the non-covalent binding of small molecules to double DNA structure by intercalation or interaction with the minor groove of nucleic acids. Based on the results of QSAR (Quantitative structure–activity relationship) studies, we propose the mode of action of designed compounds by binding to topoisomerase IIα (Topo IIα) in complex with DNA. Our study predicted their binding manner and the binding affinity towards Topo IIα by using molecular docking. It has been confirmed that Topo IIα is often overexpressed in different types of tumors, especially in the G2/M phase of the cell cycle and can be useful as a diagnostic marker [20,21]. Their inhibition leads to DNA double-strand breaks and apoptosis. To date, the FDA has approved etoposide, doxorubicin and mitoxantrone as Topo II-targeted chemotherapy agents [22].

## 2. Results and Discussion

### 2.1. Chemistry

**Scheme 1:**
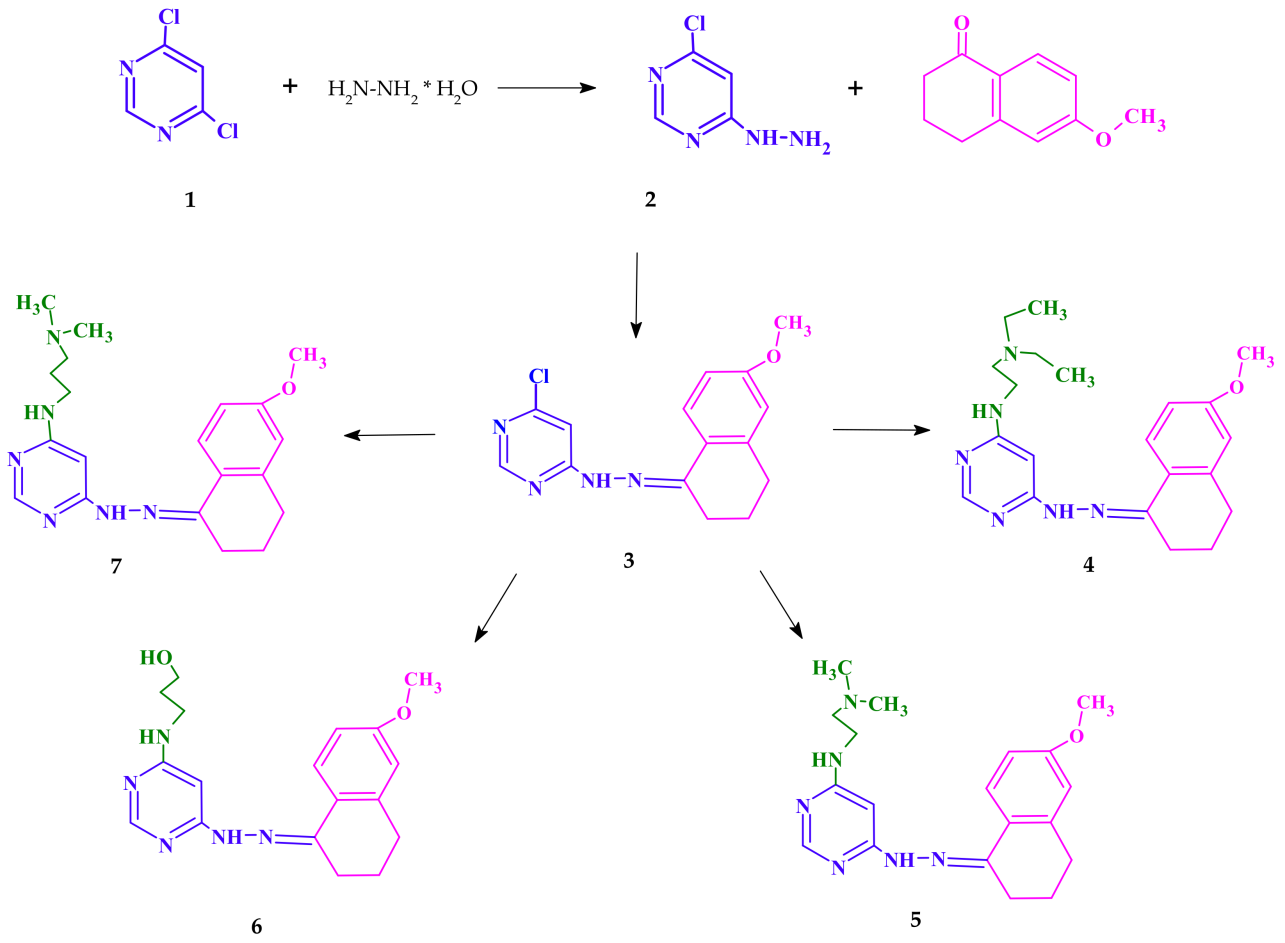
Synthesis of new pyrimidine derivatives.

The starting point of synthesis was the reaction of 4,6-dichloro-pyrimidine (1) with hydrazine hydrate at room temperature. The resulting compound 4-chloro-6-hydrazinopyrimidine (2) was dissolved in ethanol and reacted with 6-methoxy-1-tetralone to obtain 4-chloro-6-[2-(6-methoxy-3,4-dihydronaphthalen-1(2*H*)-ylidene)hydrazinyl]pyrimidine (3)**.** Compound 3 was refluxed in a nitrogen atmosphere at normal pressure with *N*,*N*-diethylethylenediamine or *N*,*N*-dimethylethylenediamine or 3-amino-1-propanol or *N*,*N*-dimethyl-1,3-propanediamine, respectively. The synthesis procedure is presented in Scheme 1 (see the Methods section for details).

### 2.2. Lipophilicity and QSAR Study

The physicochemical properties like lipophilicity, aqueous solubility, and ADME (absorption, distribution, metabolism and excretion) properties [23] are important for drug candidate substances. While absorption, distribution, metabolism and excretion are related to pharmacokinetics, lipophilicity describes drug interactions with membranes. This is a very important aspect in understanding the mechanisms of transport of substances across the membrane into the cell, especially in the context of multidrug resistance (MDR). The ADME properties could be predicted by using theoretical computational techniques which various companies widely offer. In the presented paper, theoretical physicochemical properties of doxorubicin and all the tested compounds were determined based on the Lipinski and Veber rules on the SwissADME website [24] and were collected in Table 1. According to the Lipinski’s five rules, all the parameters determined for the tested compounds were in a good agreement with parameters for substances with potentially good pharmacokinetic properties (MW ≤ 500 Da, log P ≤ 5, number of hydrogen bond donors (NHD) ≤ 5 and number of hydrogen bond acceptors (NHA) ≤ 10). 

Unfortunately, the octanol/water partition coefficient, log P_o/w_ does not exhaustively describe lipophilicity but merely quantifies the distribution of the neutral forms of the molecule instead of the distribution coefficient (log D) which includes all electrical species at a given pH. Nevertheless, log P_o/w_ is an important factor for discovering and developing new effective therapeutics [25]. Among many methods of determining the log P_o/w_ coefficient, we chose the shake flask method, which is very simple and allows us to estimate the partition coefficient's relative values in the group of compounds under study. The values obtained for doxorubicin and the synthesized compounds 4, 5 and 7 are summarized in Table 2. The UV–Vis spectra are shown in Figure S1 (Supplementary Materials).

Comparing the spectra obtained for standard doxorubicin in a buffer solution at pH 7.4 with the spectra obtained after shaking with n-octanol, the spectra of the aqueous and organic layers were very similar (Figure S1). This indicates that the form and concentration of doxorubicin were similar in both phases. It is worth mentioning that it is a simple model of an organic/water system that can only account for hydrophobic interactions. This agrees with the fact that about half of the dose is excreted unchanged from the body [27], which might suggest that it does not cross biological membranes. The concentration of the tested compound of the substances in the organic phase significantly exceeded the concentration in the aqueous phase, which is reflected in the log P_o/w_ values. This may explain why the cytotoxic efficacy of the tested compounds compared to doxorubicin was higher. The best extraction into the organic layer was observed in the case of compound 4, which was followed by compounds 7 and 5 (Figure S1).

**Figure 1:**
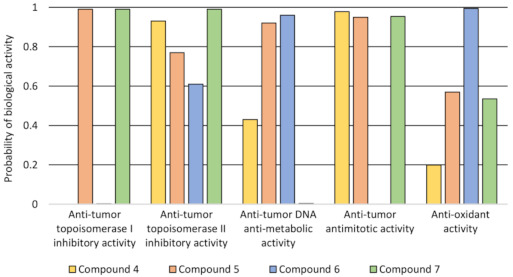
The probability of biological activity predicted based on the 3D/4D QSAR algorithm (0.0–0.2—extremely low, 0.2–0.8—moderate, 0.8–1.0—very high).

The 3D4D/QSAR model with a restricted docking protocol was also used to determine the biological activity of pyrimidine derivatives. We estimated the probability of their inhibitory activity towards topoisomerases I and II. We also analyzed anti-oxidant, DNA anti-metabolic and antimitotic activities. As presented in Figure 1, compounds 4 and 7 exhibited very high probability of activity towards the Topo II enzyme. Compounds 4, 5 and 7 had antimitotic activity. QSAR analysis also indicated that compound 6 showed high probability of having anti-oxidant and DNA antimetabolic activity.

### 2.3. Biological Evaluation

#### 2.3.1. Cytotoxicity Assay

The high mortality rate among people with cancer is due to the lack of an effective anticancer drug that selectively targets cancer cells. In this article, we present new 4,6-substituted pyrimidine derivatives with anticancer activity. For a compound to exhibit pharmacological activity, its constituent parts should exhibit pharmacological activity. Properly selected structures contribute to finding a new group exhibiting activity and selectivity and low toxicity to healthy cells. The new structures are combinations of pyrimidine–hydrazone, dihydronaphthalene and alkylamine chain groups. Pyrimidine is a well-known aromatic, heterocyclic organic compound with many different biological functions [3,28,29].

Another functional group present in biologically active agents is hydrazone. It exhibits a broad spectrum of anticancer and antimicrobial activity [14,15,16,17,18,19]. On the other hand, there are reports in the scientific literature about dihydronaphthalene showing biological activity [30,31,32].

**Figure 2:**
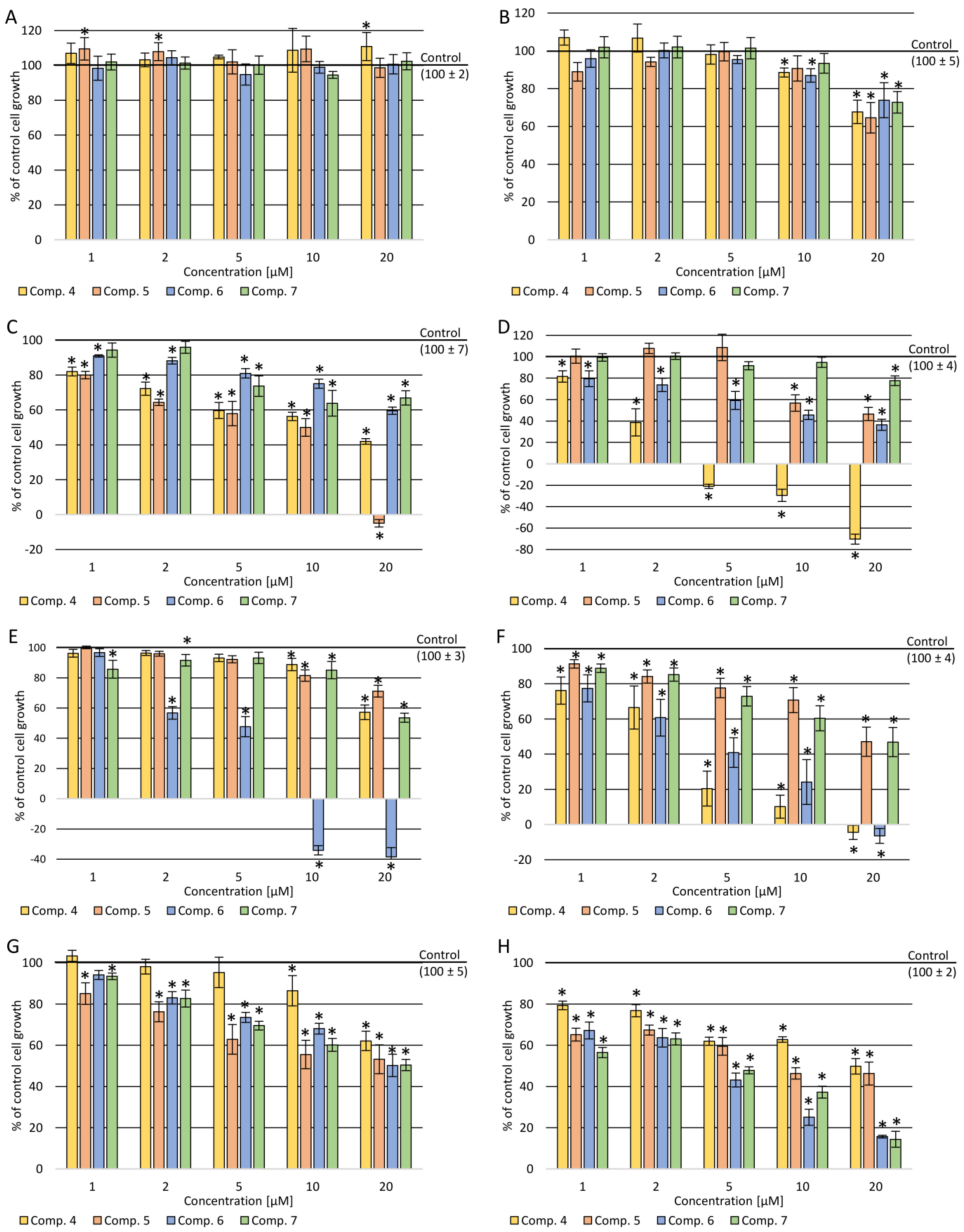
Cytotoxicity effect after incubation of cells with the tested compounds: (**A**) NHDF cells; (**B**) HeLa cells; (**C**) A549 cells; (**D**) MCF7 cells; (**E**) CCRF-CEM cells; (**F**) THP-1 cells; (**G**) LoVo cells; (**H**) LoVo/Dx cells. Data presented as a mean and SEM (standard error of the mean); * *p* < 0.05—significant difference compared to the control.

The sulforhodamine B (SRB) test was carried out on eight cell lines to characterize cytotoxic properties of the newly synthesized pyrimidine derivatives—one normal and seven cancerous. It was expected to show no cytotoxic effect on cultures of normal cells (normal human dermal fibroblasts (NHDF)) and have cytotoxic effect against tumor cells (cervical cancer (HeLa), breast cancer (MCF7), acute monocytic leukemia (THP-1), adenocarcinoma (LoVo) colorectal carcinoma cell lines—LoVo and LoVo/DX (doxorubicin-resistant), lung cancer (A549) and acute lymphocytic leukemia (CCRF/CEM)). All the tested compounds in the concentration range of 1–20 µM did not reduce the NHDF cells’ viability compared to the control (cultures without tested compounds) and the T0 control (cell culture without the tested compounds’ treatment fixed before adding compounds to the remaining culture plates). Moreover, for compound 7, a statistically significant increase in total cellular protein was observed compared to the control at a concentration of 20 µM. As shown in Figure 2B–H, after incubation with all the administered compounds, a reduction in the amount of total protein in all tumor cell cultures was present.

A decrease in the amount of total protein was observed depending on the concentration used—the higher the concentration, the stronger the cellular protein's inhibition. In the case of THP-1 culture cells, a statistically significant cytotoxic effect of compound 4 was observed in the concentration range of 5–20 µM. In the culture of CCRF-CEM cells, a cytotoxic effect of approx. 8% was demonstrated at a concentration of 20 µM of (5). In a culture of A549 cells, a statistically significant cytotoxic effect was shown in the concentration range of 10–20 µM after incubation with (6). In contrast, in the culture of MCF-7 cells, a slight cytotoxic effect was observed at a concentration of 20 µM after incubation with compounds 4 and 6. In contrast, cell growth inhibition was observed in resistant and sensitive colon cancer cells (stronger in the resistant cell line), but no cytotoxic effect.

#### 2.3.2. Assessment of the Impact on the Transport Function of P-glycoprotein—Accumulation of Rhodamine 123 (Rod-123) in Cells

Due to the stronger inhibition of the amount of cellular proteins in doxorubicin-resistant colorectal cancer cell cultures than in the counterpart of the cytostatic-sensitive cell line, the activity of P-glycoprotein was tested [11].

**Figure 3:**
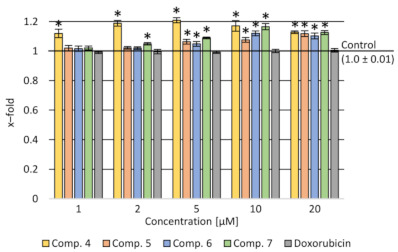
Rhodamine accumulation after incubation with the tested compounds and doxorubicin in various concentration ranges in LoVo/DX cells. Data presented as a mean and SEM (standard error of the mean); * *p* < 0.05—significant difference compared to the control.

Results show a stronger activity inhibiting the compound’s release from LoVo/DX cells compared to doxorubicin for all the tested compounds in the concentration range of 5–20 µM. It was shown that compound 4 strongly influenced the accumulation of rhodamine in cells in the whole range of concentrations tested (Figure 3).

#### 2.3.3. Verification of Apoptotic and Necrotic Cell Death

**Figure 4:**
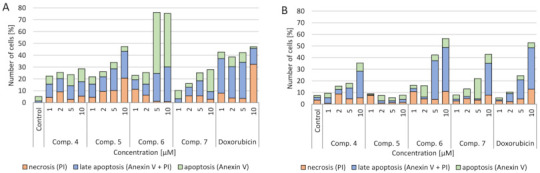
Effect of tested compounds and doxorubicin (Dox) on the level of apoptotic cells (apoptosis, late apoptosis and necrosis after 24 h of incubation): (**A**) A549 cell line; (**B**) CCRF-CEM cell line. Apoptosis—annexin V (conjugated with fluorescein) combines with phosphatidylserine, which as a result of damage is located on the outer side of the cell membrane (the color of the cell is green); late apoptosis—when the continuity of the cell membrane has been broken and propidium iodide has entered the cell (the cell has two colors—green and red); necrosis—when the membrane is degraded and the cell stains only red as a result of propidium iodide fusion with the cell nucleus.

The impact of synthesized compounds on neoplastic cell death (A549 lung cancer and CCRF-CEM leukemia) was tested. It was observed that all the tested compounds had a statistically significant effect on cell death as a result of apoptosis (both apoptosis and late apoptosis) (Figure 4). Particularly noteworthy is that compound 6 in concentrations of 5 and 10 µM increased the number of cells in the apoptotic phase to over 70%. It is about 20% more than after using the standard drug—doxorubicin. A similar but much smaller effect was seen in the CCRF-CEM cell culture. These cells are characterized by the presence of the mutant p53 protein. The increase of the number of cells in the apoptotic phase was greater at 5 and 10 µM concentrations of (6) than at the corresponding doxorubicin concentrations.

#### 2.3.4. Cell Cycle

**Figure 5:**
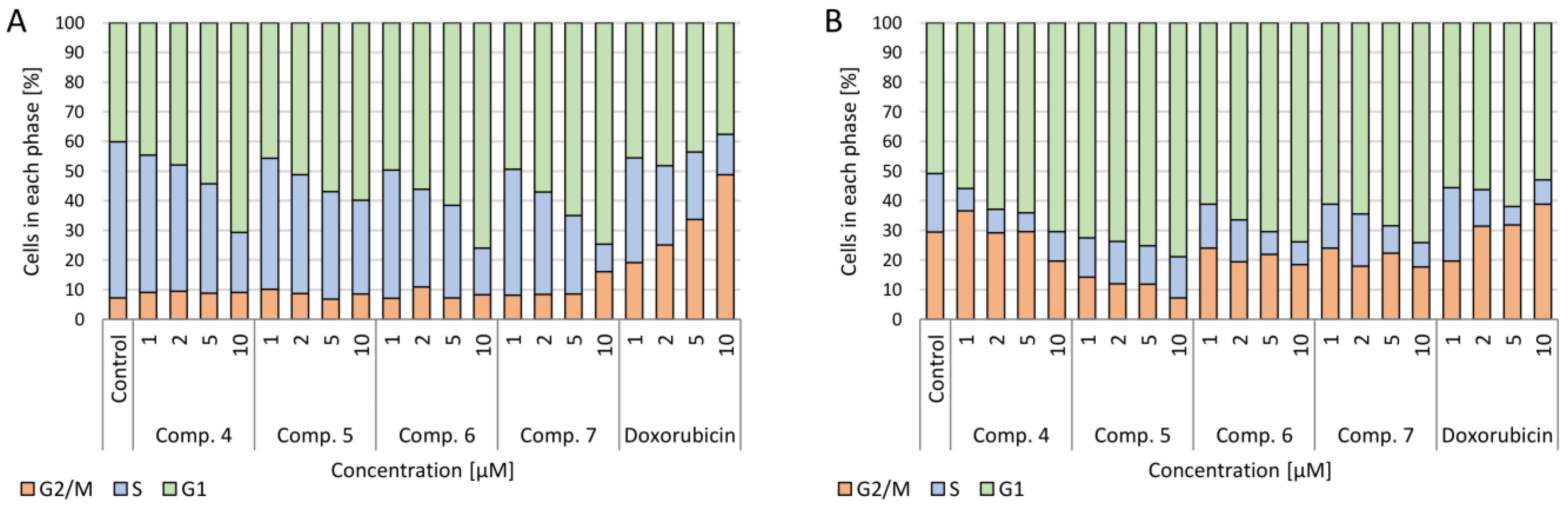
Effect of the tested compounds and doxorubicin (Dox) on the cell cycle after 24 h of incubation: (**A**) CCRF-CEM cell line; (**B**) A549 cell line.

The influence of the tested compounds on the cell cycle was also checked. In both A549 and CCRF-CEM cell cultures, a concentration-dependent increase in the number of cells in the G2/M phase after incubation with doxorubicin was observed. On the other hand, after applying the tested compounds, an increase in the number of cells in the G1 phase and a decrease in the proliferation (S) phase in both lung cancer and leukemia cells were observed (Figure 5).

#### 2.3.5. Cell Migration

**Figure 6:**
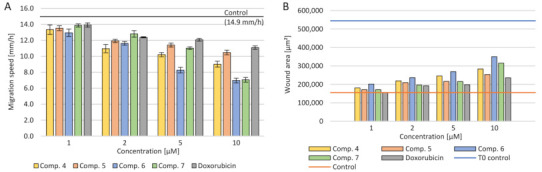
Effect of the tested compounds and doxorubicin (Dox) on the migration of cells in the scratch assay after 24 h of incubation for the A549 cell line: (**A**) migration speed; (**B**) wound area after 24 h.

A serious problem in the treatment of cancer is the risk of metastasis and rapid uncontrolled tumor growth. Therefore, reduction of the crack growth rate in the monolayer of A549 cell culture was investigated. The change increment rate was calculated during the 24-h cultivation with the tested primers (Figure 6). The concentration dependence was demonstrated—the higher the tested compounds’ concentration, the slower the neoplastic cells spread. At the same time, at the concentration of 10 µM, a smaller multiplication of tumor cells was observed than with doxorubicin. The use of 1 µM of all the tested compounds and doxorubicin did not reduce tumor progression than the control. Simultaneously, the calculated scratch surface in the monolayer after 24 h of incubation was the highest at concentrations of 10–5 µM. The strongest inhibition of the spread of neoplastic cells was observed after the use of (6). 

### 2.4. Molecular Docking

**Figure 7:**
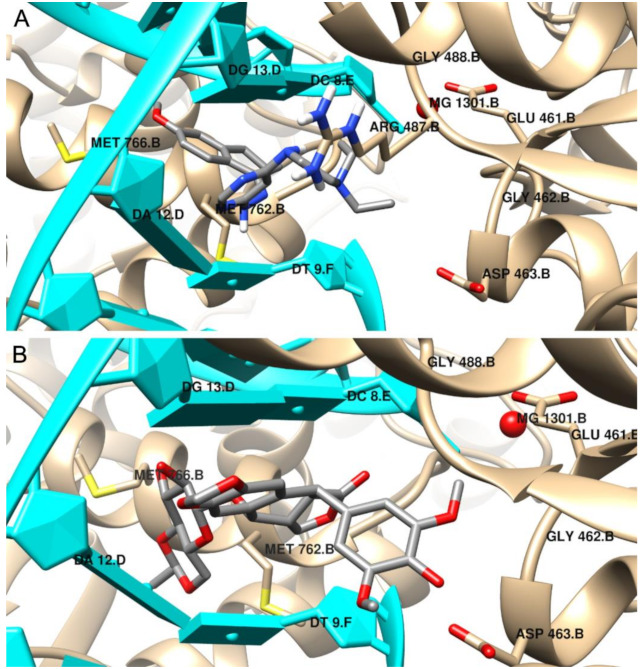
The binding mode of: (**A**) the most potent inhibitor 4, (**B**) etoposide in the active site of Topo IIα; 3D representation, DNA structure is marked in cyan, the protein chain is yellow, ligand structures are grey.

**Figure 8:**
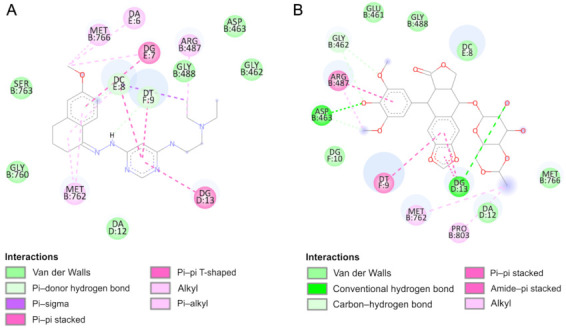
Intermolecular interactions in the active site of Topo IIα (2D representation) of: (**A**) the most potent inhibitor 4, (**B**) etoposide.

We performed a molecular docking study to determine the binding mode of synthesized compounds to the Topo II/DNA complex (PDB ID 5GWK). We obtained the free energy of binding (ΔG_binding_) and the inhibition constants (K_i_) (see Table 3). Additionally, the non-covalent interactions were characterized in detail. The docking protocol was validated by docking the co-crystalized ligand etoposide to the active site of Topo IIα. Our results are consistent with the earlier studies [33,34]. Etoposide binds to the binding site of topoisomerase and DNA with the free energy of binding equal to –44.6 kJ/mol. The aglycone part of the inhibitor localized between nucleic acid bases is involved in drug–DNA interactions (π–π interactions with thymine DT9 and guanine DG13). The podophyllotoxin moiety also contributes to drug–protein interactions and binds to the pocket created by Glu461, Gly462, Asp463, Arg487 and Gly488. The complex is also stabilized by van der Waals interactions and hydrogen bonds created with Gly462, Asp463 and guanine DG13 (see Figure 7 and Figure 8 and Supplementary Figure S2).

According to the results, the most potent inhibitor of Topo IIα is compound 4 (inhibition constant, 4.12 µM). The pyrimidine ring is intercalated in double-stranded DNA and interacts via π–π- interactions with cytosine DC8, thymine DT9 and guanine DG13. The naphthalene moiety is involved in π-stacked interactions with guanine DG7 and π–alkyl interactions with Met762. Additionally, compound 4 forms van der Waals interactions with a number of polar, charged and hydrophobic amino acids of topoisomerase, namely, Gly462, Asp463, Gly488, Gly760 and Ser763.

As can be observed, the geometry of the designed ligands affects their binding properties and inhibitory activity. The data are presented in Table 3 and in the Supplementary Materials. The *N*,*N*-diethylethylenediamine group with a *N*,*N*-dimethylethylenediamine fragment slightly changes the nature of interactions (compound 5). In this case, the naphthalene ring interacts via π–π stacking interactions with cytosine DC8, thymine DT9 and guanine DG13 and via π–σ interactions with adenine DA12. The van der Waals interactions of Glu461, Glu462, Gly760, Met762 and Tyr805 amino acid residues of Topo II and pyrimidine and *N*,*N*-dimethylethylenediamine fragments are present. The total intermolecular interaction energy for this complex is equal to −37.7 kJ/mol.

The results indicate that compound 6 is the weakest inhibitor of topoisomerase (ΔG_binding_ = −26.3 kJ/mol), which is consistent with the quantitative structure–activity relationship study. It is probably related to the replacement of the branched chain of the 3-amino-1-propanol group, which interacts with the protein via hydrogen bonds with Asp463 and van der Waals interactions with Gly462, Ser464 and Gly488 amino acid residues. The naphthalene moiety exhibits a unique binding configuration, namely, interacts through π–σ interactions with guanine DG13, via a π–π stacked configuration with thymine DT9 and via π–alkyl interaction with adenine DA12. Its mechanisms of action can probably be different. QSAR studies showed that it has potential anti-oxidant and DNA antimetabolic activity. On the other hand, an in vitro study showed its strong proapoptotic properties.

Compound 7 can form hydrogen bonds with guanine molecules (DG10 and DG13). Additionally, the naphthalene ring is directly exposed to π–π interactions with thymine DT9 and guanine DG13. One alkyl interaction was observed between *N*,*N*-dimethyl-propanediamine chain and the Lys440 residue. In this case, three carbon–hydrogen bonds and van der Waals interactions were detected with Asp463, Pro485, Glu506 and Gly462 and Leu486, respectively.

## 3. Conclusions

Our study revealed many differences in the responses of cell lines to newly synthesized pyrimidine derivatives. These data suggest that the compounds tested may represent an antitumor potential which may vary with the tumor type. According to the results, the tested compounds may be beneficial in the treatment of doxorubicin-resistant neoplasms. Lipophilicity studies may suggest that a higher affinity to the 1-octanol phase increases the probability of penetration of the tested compounds into the cancer cells. As indicated in the literature, Topo IIα is often overexpressed in different types of tumors cells, especially in the G2/M phase of the cycle. Their inhibition leads to DNA double-strand breaks and apoptosis. Our data suggest that the proposed compounds are capable of inhibit the activity of Topoisomerase IIα and intercalate DNA. Additionally, all derivatives can decrease the number of cells in the proliferation (S) and G2/M phase. All exhibit proapoptotic properties. In conclusion, more detailed studies are needed to determine the tested compounds’ mechanism of action of towards tumor cells and their structures should be further optimized, before they can be considered anticancer agents.

## 4. Materials and Methods 

### 4.1. Instruments and Materials

All the reagents and solvents were used without further purification. Melting points were determined by an LLG uniMELT-2 apparatus (LLG). Column chromatography was carried out on silica gel (Merck Kieselgel 100). Progress of the reaction was controlled by thin-layer chromatograph SiliaPate TLC Aluminium Backed TLC (Silicycle UltraPure Silica Gels, Québec, Canada) and visualized using ultraviolet (UV) light at 254 nm and 365 nm (Vilbe Lourmat, Collégien, France). ^1^H NMR (300.14 MHz) and ^13^C NMR (75.4 MHz) were recorded on a Bruker ARX-300 spectrometer (Bruker Analytische Messtechnik GmbH, Rheinstetten, Germany). Multiplicities of NMR signals were designated as follows: s (singlet), d (doublet), t (triplet), m (multiplet). Mass spectrometry (MS) was performed on a compact^TM^ Electrospray Ionization–Quadrupole Time-of-Flight (ESI–Q-TOF) apparatus (Bruker Daltonics, Billerica, MA, USA). All the NMR and MS measurements were carried out in the Laboratory of Elemental Analysis and Structural Research, Faculty of Pharmacy, Wroclaw Medical University.

### 4.2. Synthesis

4-Chloro-6-hydrazinopyrimidine (**2**)

4,6-Dichloropyrimidine (1) (6 g, 40 mmol) was dissolved in 30 mL of ethanol, and 8 mL hydrazine hydrate was added. The mixture was stirred at room temperature for 1 h. The precipitate was filtered off. The solid was taken up to the water (50 mL). The residue was filtered off. Yield: 88%; m.p. 168 °C. ^1^H NMR (DMSO-*d*_6_): δ 4.45 (s, 2H, -NH_2_), 6.73 (s, 1H, NH), 8.15 (s, 1H, 2-H), 8.77 (s, 1H, 5-H). ESI–MS (*m*/*z*): calc. for C_4_H_5_ClN_4_ [M + H]^+^, 145.0275; found, 145.0275 (see Figure S3).

4-Chloro-6-[2-(6-methoxy-3,4-dihydronaphthalen-1(2*H*)-ylidene)hydrazinyl]pyrimidine (**3**)

A mixture of compound 2 (1 g, 6 mmol), 70 mL ethanol and (1.3 g, 7 mmol) 6-methoxy-1-tetralone were refluxed with stirring for 3 h. The resulting precipitate was filtered off and recrystallized from ethanol to give 1.7 g of compound 6. Yield: 91%; m.p. 140 °C. ^1^H NMR (300 MHz, DMSO-*d*_6_): δ 1.81 (m, 2H, 2x 3-H), 2.68 (m, 4H, 2x 2-H, 2x 4-H), 3.76 (s, 3H, -OCH_3_), 6.75 (s, 1H, 5-H), 6.82 (d, 1H, 7-H), 7.17 (s, 1H, 5-H pyrimidine), 8.05 (d, 1H, 8-H), 8.41 (s, 1H, 2-H pyrimidine), 10.67 (s, 1H, NH) (see Figure S4). ^13^C NMR (75.4 MHz, DMSO-*d*_6_): δ 163.47 (C4 pyrimidine), 160.41 (C2 pyrimidine), 159.78 (C1’), 158.63 (C5 pyrimidine), 150.40 (C6’), 142.05 (C8’), 126.76, 125.37 (C4’a, C8’a), 113.82 (C7’), 112.90 (C5’), 102.34 (C6 pyrimidine), 55.60 (OCH_3_), 29.61 (C2’), 26.20 (C3’), 21.84 (C4’) (see Figure S5). ESI–MS (*m*/*z*): calc. for C_15_H_15_N_4_OCl [M + H]^+^, 303.1007; found, 303.0991 (see Figure S6). 

General Procedure for the Synthesis of Compounds 4, 5, 6, 7

A mixture of compound 3 (0.2 g, 0.6 mmol) and *N*,*N*-diethylethylenediamine (10 mL) (*N*,*N*-dimethylethylenediamine (10 mL), 3-amino-1-propanol (10 mL) or *N*,*N*-dimethyl-1,3-propanediamine (10 mL)) and a few drops of DMF (*N*,*N*-dimethylformamide) were refluxed in the nitrogen atmosphere at normal pressure for 2 h. After evaporation to dryness, the residue was purified by column chromatography on a silica gel column and eluted with methylene chloride/methanol (99:1).

*N*-{6-[2-(6-Methoxy-3,4-dihydronaphthalen-1(2*H*)-ylidene)hydrazinyl]pyrimidin-4-yl}-*N*’*N*’-diethylethane-1,2-diamine (**4**)

Yield: 0.10 g (39%); m.p. 127 °C. ^1^H NMR (DMSO-*d*_6_): δ 0.97 (m, 6H, 2x CH_3_), 1.80 (m, 2H, 2x3-H), 2.16 (s, 3H, CH_3_), 2.51 (m, 4H, 2x4-H, 2x2-H,),), 2.62 (m, 4H, 2xCH_2_), 3.29 (m, 4H 2xCH_2_, 3.75 (s, 3H, OCH_3_), 6.20 (s, 1H, NH), 6.72 (m, 3H, 5-H pyrimidine 7-H, 5-H), 7.97 (m, 2H, 8-H, 2-H pyrimidine), 9.44 (s, 1H, NH) (see Figure S7). ^13^C NMR (75.4 MHz, DMSO-*d*_6_): δ 163.53 (C4 pyrimidine), 159.80 (C2 pyrimidine), 157.80 (C5 pyrimidine), 146.09 (C6’), 141.25 (C8’), 126.20, 125.94 (C4’a, C8’a), 113.56 (C7’), 112.58 (C5’), 55.57 (OCH_3_), 52.05 (C6 pyrimidine), 47.17 (CH_2_), 29.70 (C2’), 25.61 (C3’), 21.86 (C4’), 12.23 (CH_3_) (see Figure S8). ESI–MS (*m*/*z*): calc. for C_21_H_30_N_6_O [M + H]^+^, 383.2553; found, 383.2529 (see Figure S9).

*N*-{6-[2-(6-Methoxy-3,4-dihydronaphthalen-1(2*H*)-ylidene)hydrazinyl]pyrimidin-4-yl}-*N*'*N*'-dimethylethane-1,2-diamine (**5**)

Yield: 0.06 g (26%); m.p. 189 °C. ^1^H NMR (DMSO-*d*_6_): δ 1.79 (m, 2H, 2x3-H), 2.16 (s, 3H, CH_3_), 2.40 (t, 2H, 2x2-H,), 2.48 (t, 2H, 2x4-H), 2.62 (t, 2H, CH_2_), 2.69 (t, 2H, CH_2_), 3.75 (s, 3H, OCH_3_), 6.23 (s, 1H, NH), 6.77 (m, 3H, 5-H pyrimidine 7-H, 5-H), 7.98 (m, 2H, 8-H, 2-H pyrimidine), 9.45 (s, 1H, NH) (see Figure S10). ^13^C NMR (75.4 MHz, DMSO-*d*_6_): δ 163.45 (C4 pyrimidine), 161.90 (C1’) 159.79 (C2 pyrimidine), 157.78 (C5 pyrimidine), 146.04 (C6’), 141.23 (C8’), 126.21, 125.95 (C4’a, C8’a), 113.59 (C7’), 112.83 (C5’), 58.45 (C6 pyrimidine), 55.57 (OCH_3_), 45.40 (CH_3_), 29.70 (C2’), 25.60 (C3’), 21.86 (C4’) (see Figure S11). ESI–MS (*m*/*z*): calc. for C_19_H_26_N_6_O [M + H]^+^, 355.2240; found, 355.2225 (see Figure S12).

3-({6-[2-(6-Methoxy-3,4-dihydronaphthalen-1(2*H*)-ylidene)hydrazinyl]pyrimidin-4-yl}amino)propan-1-ol (**6**)

Yield: 0.07 g (31%); m.p. 182 °C. ^1^H NMR (DMSO-*d*_6_): δ 1.69 (m, 2H, CH_2_), 1.79 (m, 2H, 2x 3-H), 2.61 (t, 2H, 2x2-H), 2.69 (t, 2H, 2x4-H), 3.29 (m, 6H, 3xCH_2_), 3.75 (s, 3H, OCH_3_), 4.48 (t, 1H, OH), 6.18 (s, 1H, NH), 6.73 (s, 1H, 5-H), 6.77 (d, 1H, 7-H), 6.79 (s, 1H, 5-H pyrimidine), 7.95 (d, 1H, 8-H), 7.99 (s, 1H, 2-H pyrimidine), 9.42 (s, 1H, NH) (see Figure S13). ^13^C NMR (75.4 MHz, DMSO-*d*_6_): δ 163.63 (C4 pyrimidine), 159.78 (C2 pyrimidine), 157.79 (C5 pyrimidine), 146.10 (C6’), 141.22 (C8’), 126.21, 125.96 (C4’a, C8’a), 113.60 (C7’), 112.86 (C5’), 58.94 (C6 pyrimidine, CH_2_), 55.57 (OCH_3_), 29.70 (C2’), 25.61 (C3’), 21.87 (C4’) (see Figure S14). ESI–MS (*m*/*z*): calc. for C_18_H_23_N_5_O_2_ [M + H]^+^, 342.1924; found, 342.1906 (see Figure S15).

*N*-{6-[2-(6-Methoxy-3,4-dihydronaphthalen-1(2*H*)-ylidene)hydrazinyl]pyrimidin-4-yl}-*N*'*N*'-dimethylpropane-1,3-diamine (**7**)

Yield: 0.18 g (81%); m.p. 165 °C. ^1^H NMR (DMSO-*d*_6_): δ 1.79 (m, 2H, 2x 3-H), 2.27 (s, 6H, 2xCH_3_), 2.69 (m, 6H, CH_2_, 2x 2-H, 2x 4-H), 3.28 (m, 4H, 2xCH_2_), 3.74 (s, 3H, OCH_3_), 6.19 (s, 1H, NH), 6.72 (s, 1H, 5-H), 6.75 (d, 1H, 7-H), 7.07 (s, 1H, 5-H pyrimidine), 7.95 (d, 1H, 8-H), 8.02 (s, 1H, 2-H pyrimidine), 9.46 (s, 1H, NH) (see Figure S16). ^13^C NMR (75.4 MHz, DMSO-*d*_6_): δ 163.48 (C4 pyrimidine), 161.87 (C1’) 159.81 (C2 pyrimidine), 157.77 (C5 pyrimidine), 146.23 (C6’), 141.27 (C8’), 126.14, 125.96 (C4’a, C8’a), 113.59 (C7’), 112.84 (C5’), 55.76 (CH_2_), 55.58 (OCH_3_), 44.49 (C6 pyrimidine), 43.54 (CH_3_), 29.68 (C2’), 25.62 (C3’), 21.86 (C4’) (see Figure S17). ESI–MS (*m*/*z*): calc. for C_20_H_28_N_6_O [M + H]^+^, 369.2397; found, 369.2394 (see Figure S18).

### 4.3. Lipophilicity and QSAR Studies

The shake flask method was used to experimentally determine the value of log P_o/w_ for all three compounds (4, 5 and 7). All experiments were run in the aqueous (50 mM HEPES, pH 7.4 and *I*_KCl_ = 0.16 M, 25 °C) and 1-octanol solvents. The following were mixed: 0.75 mL of 1-octanol and 0.75 mL of a solution of the studied substances. The tested compounds were dissolved in a small amount of DMSO, and the buffer was added, so the final concentration of DMSO in measured samples was less than 0.2%. The concentrations of used solutions were as follows: doxorubicin = 5 × 10^−5^ M and compounds 4, 5, 7: 2 × 10^−5^ M, 1.5 × 10^−5^ M and 1.8 × 10^−5^ M, respectively. The compound 6 (2 × 10^−5^ M) solution precipitated after buffer addition, so it was not used for further studies. The samples were shaken manually (∼2 min), vortexed (∼5 min) and centrifuged (∼3 min, 6000 rpm). Both layers were carefully separated and measured by UV–Vis spectroscopy using 1-cm quartz cuvettes. The Beer–Lambert law was used to determine the ligand and complex concentration in the analyzed layer (*ε* and *λ*_max_ are given in Table 4). The measurements were run in triplicate.

### 4.4. Tested Compounds

Newly synthesized compounds were dissolved in DMSO to a 10 mM concentration, and these stocks were stored at −20 °C for up to one month. In biological studies, the compounds were used in a concentration range of 1–20 µM by dissolving the stock solution with a complete medium appropriate to the cell lines (so DMSO concentration did not exceed 0.2%). 

#### 4.4.1. Cell Lines and Conditions

All biological assays were performed on eight cell lines: one normal and seven cancerous ones. Normal human dermal fibroblasts (NHDF) were cultured in the DMEM without phenol red. The colorectal carcinoma cells (LoVo) were grown in DMEM-F12. The breast tumor (MCF-7), lung cancer (A549), and cervical cancer cells (HeLa) were incubated in the MEM. The human leukemic lymphoblasts (CCRF-CEM) and human monocytic cells (THP-1) were cultured in RPMI-1640. All the media were supplemented with 10% FBS, 2 mM l-glutamine and 25 μg/mL gentamicin. All cell lines were grown at 5% CO_2_, 95% humidity, 37 °C and their morphology and confluency were assessed under a microscope minimum twice a week. When confluency was greater than 70%, adherent cell lines were detached from the bottle's surface with a TrypLE solution and then reduced or used in assays. The cells growing in suspension were reduced or also used in biological assays. After each cell line’s subculture, the cells were collected into centrifuge tubes and centrifuged at 1000× *g* for 5 min. The supernatant was removed, and the cell pellet was resuspended in an appropriate medium. The cells were counted using the Bürker chamber, and then the cells were resuspended again to a cell density of 10,000 cells per well. Ninety-six-well plates were used for the MTT assay, whereas 24-well plates were used for dual-fluorescence viability analysis. The cells thus seeded were incubated overnight under standard conditions (5% CO_2_, 95% humidity, 37 °C) for cell adherence to the well surfaces and cell regeneration. The next day, the concentration of the tested compounds was prepared and added to the plate so that the final concentration in the culture plates was 1, 2, 5, 10 and 20 µM with the cells for 24 h in MTT assay.

#### 4.4.2. Viability Assay

To evaluate the tested compounds’ influence on the viability of the used cell lines, the MTT assay was performed. For the MTT assay, 20,000 cells per well of adherent lines (NHDF, HeLa, LoVo, MCF7, LoVo/DX, A549) were seeded, as well as 40,000 cells per well of suspension lines (CCRF-CEM and THP-1). After incubating cells with the tested compounds, the medium was removed from the adherent cells. A freshly prepared MTT solution (1 mg/mL tetrazolium salt in the MEM without phenol red) was added for 2 h at 37 °C. Next, the supernatant was removed, and formazan crystals were dissolved in isopropanol by shaking for 30 min. The MTT solution (5 mg/mL tetrazolium salt in the MEM without phenol red) was added to the cells growing in suspensions immediately after 24 h of incubation with the tested compounds and left at 37 °C, but for four hours. In the next step, the lysis buffer was added and left overnight. Finally, after dissolving the crystal in the adherent cells and lysing the cells growing in suspension, the absorbance was measured at 570 nm using a Varioskan LUX microplate reader (Thermo Fisher Scientific, Waltham, MA, USA).

#### 4.4.3. Assessment of the Impact on the Transport Function of P-glycoprotein—Accumulation of Rhodamine 123 (Rod-123) in Cells

To assess the effect on the transport functions of P-glycoprotein (P_gp_), a test of accumulation of rhodamine 123 (Rod-123) in cells was performed. A Rod-123 (Sigma, Burlington, MA, USA) solution at a 10 mM concentration in a 1:1 DMSO/water mixture was prepared before the experiment.

The cells were seeded in sterile opaque-walled 96-well plates in 100 µL culture medium and 2 × 10^4^ cells/well of LoVo doxorubicin-resistant cells and grown for 24 h. At the beginning of the experiment, solutions of the tested compounds in the DMEM F-12 medium (without FBS) were added to the test wells at a volume of 100 µL/well. The cells were incubated with the tested compounds for two hours. After this time, Rod-123 was added to the wells to a final concentration of 12.5 µM and incubated for 60 min. After incubation, the supernatant was removed. The cells were dissolved (150 µL/well) in 20 mM Tris-HCl (pH 7.7) containing 0.2% sodium dodecyl sulfate (SDS) to lyse the cells and release the intracellular fluorescent substrate. Fluorescence measurement was performed with the use of a Varioskan LUX excitation reader (ex. 485 nm, em. 538 nm). 

#### 4.4.4. Verification of Apoptotic and Necrotic Cell Death

To assess the number of cells (A549 and CCRF-CEM cell lines) in apoptosis and necrosis after treatment with the tested compounds, cell culture staining with annexin V-conjugated fluorescein and propidium iodide was performed. Eighty thousand A549 cells per well and 160,000 CCRF-CEM cells per well in 24-well plates were seeded to assess the number of cells in apoptosis and necrosis. After 24 h of incubating cells with the tested compounds, the medium was transferred from the adherent cells to the previously prepared tubes, and the TrypLE solution was added into wells for 3 min at 37 °C. After detaching, the cells were transferred into tubes with the earlier collected medium. The CCRF-CEM cells, which were growing in suspension, were transferred into tubes immediately after 24-h of incubating with the tested compounds. Both cell lines were centrifuged at 1000× *g* for 5 min at RT. In the next step, the supernatant was removed, and a mixture (annexin V diluted with a buffer containing Ca^2+^ and propidium iodide) was added for 20 min in the dark at RT. The cells were then centrifuged at 1000× *g* for 5 min. After removing the supernatant, the cell pellet was resuspended in PBS, and then the suspension cells were transferred into a chip and analyzed in a CountStar counter (ALIT Life Science, Shanghai, China).

#### 4.4.5. Cell Cycle

To determine how many cells are in each of the different phases of the cell cycle, cell cultures (A549 and CCRF-CEM) were stained with propidium iodide. For cell cycle assessment, cell cultures were harvested into appropriate tubes according to the procedure described in Section 4.4 for the type of cell death (apoptosis and necrosis). After removing the supernatant, ice-cold 100% ethanol was added to the cells and centrifuged at 1000× *g* for 5 min at 4 °C. After removing the supernatant, the propidium iodide solution was added for 5 min, and the cells were centrifuged at the same condition. After suspension with PBS, the cells were transferred into a chip and analyzed in a CountStar.

#### 4.4.6. Cell Migration

To evaluate the effect of the tested compounds on tumor metastasis, a migration assay was performed on the A549 cell line. After the monolayer of cell cultures on plates was reached, a scratch was made in the culture. The tested compounds were then added to the cell cultures. Photographs were taken with an EVOS FL microscope (Thermo Fisher Scientific, Waltham, MA, USA), and then the cell cultures were incubated for 24 h, and the scratch was re-photographed. Using the ImageJ open software platform, the scratch length was measured after its preparation and 24 h after incubation with the tested compounds.

## 4.5. Statistical Analysis

All data are presented as the means ± SEM. The one-way ANOVA and appropriate (Scheffe) post-hoc test were used to calculated statistical significance. Statistical significance was set at *p* < 0.05.

## 4.6. Molecular docking

All the tested compounds were optimized using the semiempirical PM6 method of the Gaussian 09 package [37]. The calculations were performed taking into account the water solution by using the polarizable continuum model (PCM) [38,39]. Molecular docking studies were performed using the AutoDock 4.2 package and a standard protocol was followed to predict the binding mode and the free energy of binding [40]. As an input, we used a specially prepared crystal structure of Topo IIα (PDB ID 5GWK) downloaded from the Protein Data Bank (PDB) [41]. The validation was performed by docking etoposide into the crystal structures of topoisomerase and comparing its position in the original crystallographic structure. To the protein polar hydrogens, Gasteiger charges and solvent parameters were added. The water molecules and the complexed etoposide inhibitor were removed. Each of the chains of the crystal structure of Topo IIα have two Mg^2+^ ions, which are about 26.18 Å away from each other. Only one ion exhibited direct contact to protein side chains (carboxyl groups of Asp139 and Asp141) and was included in the docking grid. The Mg^2+^ ions used force field potentials as defined in the AutoDock 4.2 program (http://autodock.scripps.edu/resources/parameters/AD4.1_bound.dat/view, accessed on 1 March 2021). The charges of metals ions were assigned manually by editing the PDBQT file. According to the earlier studies, the assigned partial charge was established as +0.8 [42]. The binding site was defined using a grid of 70 × 70 × 70 points with a grid space of 0.375 Å. The center of the box was located on the active site according to crystallized inhibitor coordination. Lamarckian genetic algorithm with local search was employed with a total of 200 runs for each binding site. Previous studies showed that it is the most efficient and reliable algorithm of AutoDock 4.2 [42]. In each calculation, the population of 150 individuals with 27,000 generations and 250,000 energy evaluations was adopted. The obtained results were visualized using a Chimera and a BIOVIA Discovery Studio visualizer [43].

## Figures and Tables

**Table 1:** The drug similarity parameters were theoretically obtained from the SwissADME website [24]. ^a^ NHD—number of hydrogen bond donor; ^b^ NHA—number of hydrogen bond acceptors; ^c^ NBR—number of rotatable bonds; ^d^ TPSA—total polar surface area.

Compound	Lipinski’s Rules					Veber’s Rules	
	MW ≤ 500	Log P ≤ 5	NHD ≤ 5	NHA ≤ 10	Violations of Rules	NBR ≤ 10	TPSA ≤ 140
Doxorubicin	543.52	0.52	6	12	3	5	206.07
4	382.50	3.14	2	5	0	9	74.67
5	354.45	2.47	2	5	0	7	74.67
6	341.41	2.45	3	5	0	7	91.66
7	368.48	2.77	2	5	0	8	74.67

**Table 2:** Experimentally determined log P_o/w_ values. Shake flask method [26]; P_o/w_ = [compound]1-oktanol/[compound]HEPES; pH = 7.4; T = 25 °C; concentration determined by UV–Vis absorption. Values of consensus log P_o/w_ were determined based on the Lipinski and Veber rules on the SwissADME website.

Compound	Log P_o/w_	Consensus log P_o/w_
Doxorubicin	–0.18	0.52
4	1.05	3.14
5	1.53	2.47
7	1.25	2.77

**Table 3:** Free energy of binding, inhibition constant and intermolecular interaction energy components evaluated during molecular docking of one molecule of the inhibitor to the active site of Topo IIα.

Compound	ΔG_binding_ (kJ/mol)	K_i_ (µM)	ΔG_int_ (kJ/mol)	ΔG_vdw_ + ΔG_hbond_ + ΔG_desolv_ (kJ/mol)	ΔG_el_ (kJ/mol)
4	−30.8	4.12	−42.0	−41.6	−0.4
5	−29.0	8.56	−37.7	−37.5	−0.2
6	−26.3	24.36	−36.3	−35.5	−0.8
7	−27.0	15.77	−37.0	−36.8	−0.2
Etoposide	−44.6	0.02	−50.9	−50.6	−0.3

**Table 4:** Experimentally measured molar extinction coefficients (ε) and absorbance maxima (λ_max_) for doxorubicin and compounds 4, 5, 7 with UV–Vis-determined concentration. Solvent: HEPES buffer (50 mM, I = 0.16 M). Path length = 1 cm. T = 25 °C.

Compound	λ_max_ (nm)	ε (M^−1^cm^−1^)
Doxorubicin	539	4459
4	345	15,326
5	328	50,465
7	328	20,430

The simulations of biological properties were performed using a combination of the 3D/4DQSAR BiS/MC and CoCon algorithms developed by ChemoSophia [35,36].

## Data Availability

The data generated and analyzed during the current study are available from the corresponding authors upon reasonable request.

## Supplementary Materials

The following are available online at https://www.mdpi.com/article/10.3390/ijms22083825/s1.
