# Supplementary material for: Novel Pyrimidine Derivatives as Potential Anticancer Agents: Synthesis, Biological Evaluation and Molecular Docking Study
Source: Int J Mol Sci. 2021 Apr 7;22(8):3825. doi: 10.3390/ijms22083825 (PMC8067809; doi:10.3390/ijms22083825)
Supplement: Supplementary file 1 [file ijms-22-03825-s001.pdf]

International Journal of
Molecular Sciences

MDPI

Novel Pyrimidine Derivatives as Potential Anticancer Agents: Synthesis, Biological Evaluation and Molecular Docking Study

Beata Tylińska, Benita Wiatrak, Żaneta Czyżnikowska, Aneta Cieśla-Niechwiadowicz, Elżbieta Gębarowska and Anna Janicka-Kłos

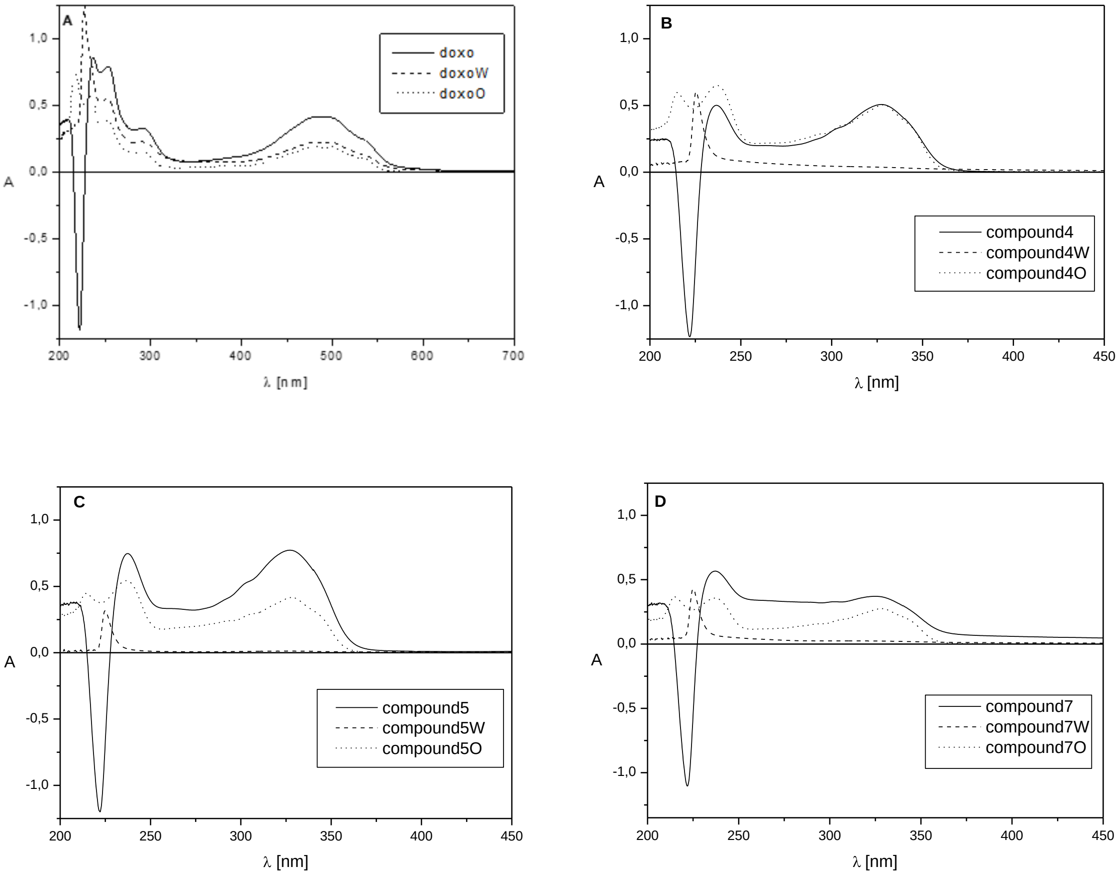

Figure S1. UV-Vis spectra of doxorubicin and tested compounds **4, 5** and **7** in HEPES buffer -water (W) and 1-octanol (O) phase. Concentration of used solutions were: doxorubicin = 5×10^{-5} M and compounds 2×10^{-5} M, 1.5×10^{-5} M and 1.8×10^{-5} M respectively. Path length = 1 cm. T = 25 °C.

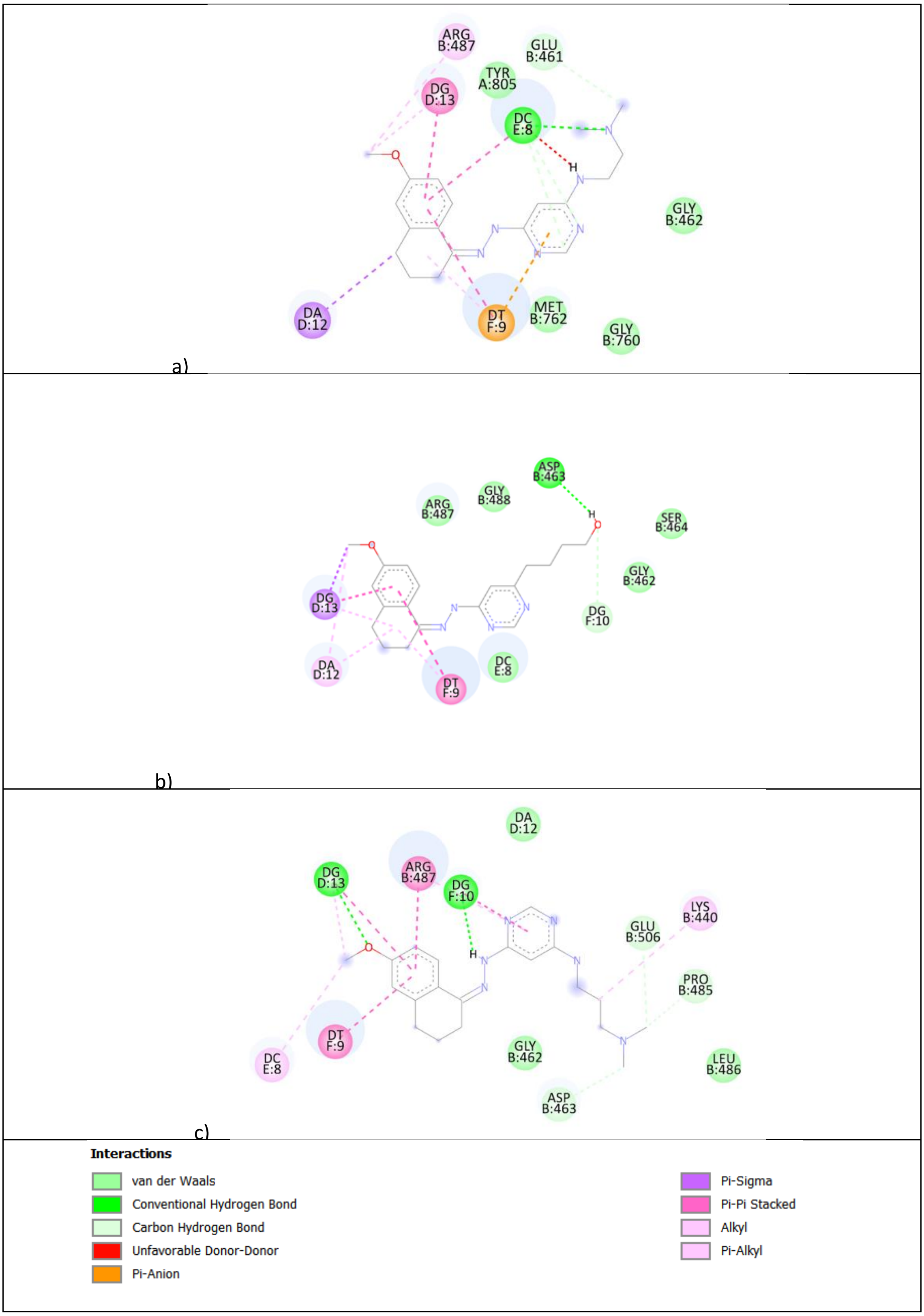

Figure S2. Intermolecular interactions in the active site of Topo IIα (2D representation): **a)** compound **5**; **b)** compound **6**; **c)** compound **7**.

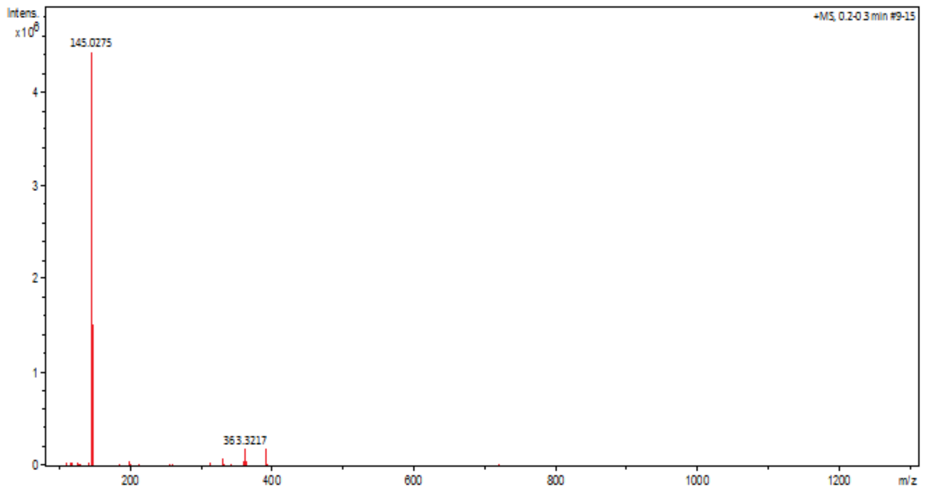

Figure S3. ESI-MS spectrum of compound **2**.

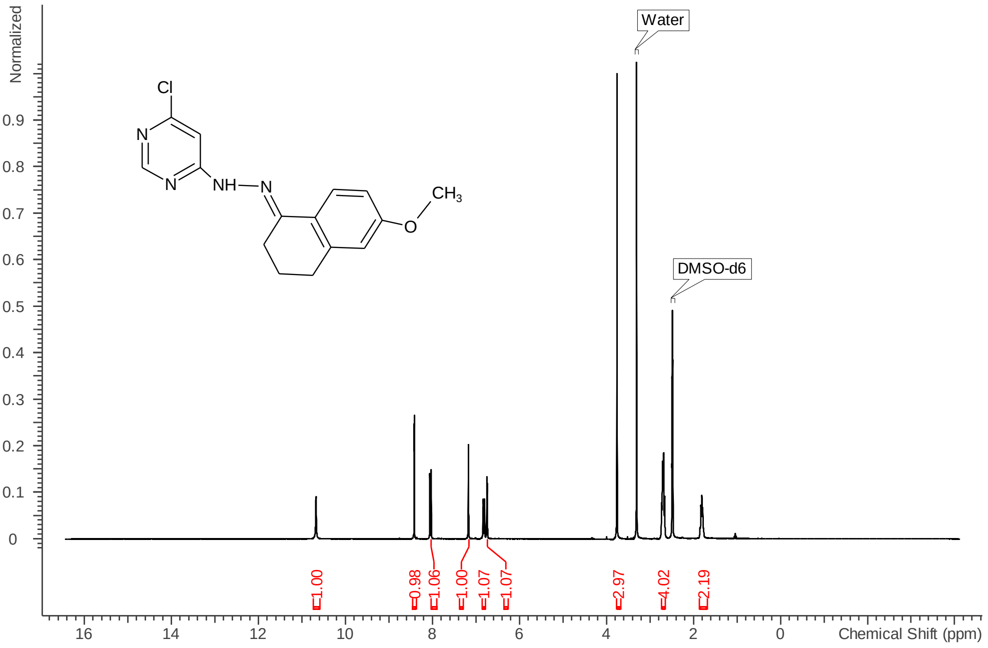

Figure S4. ^{1}H NMR spectrum of compound **3**.

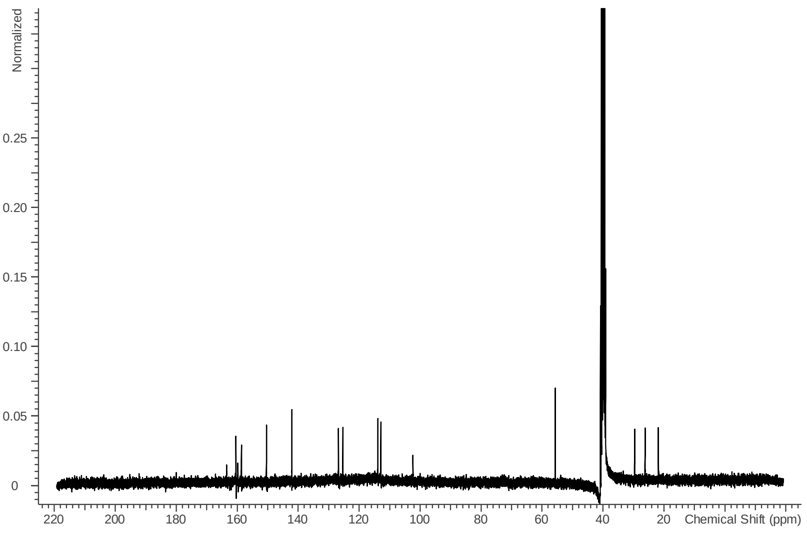

Figure S5. ^{13}C NMR spectrum of compound **3.**

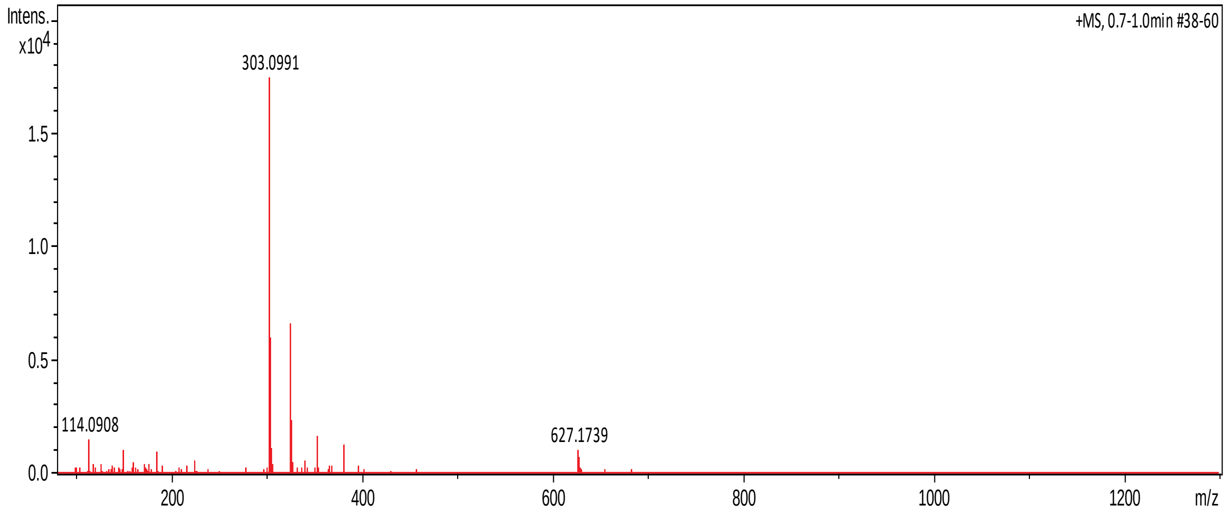

Figure S6. ESI-MS spectrum of compound **3**.

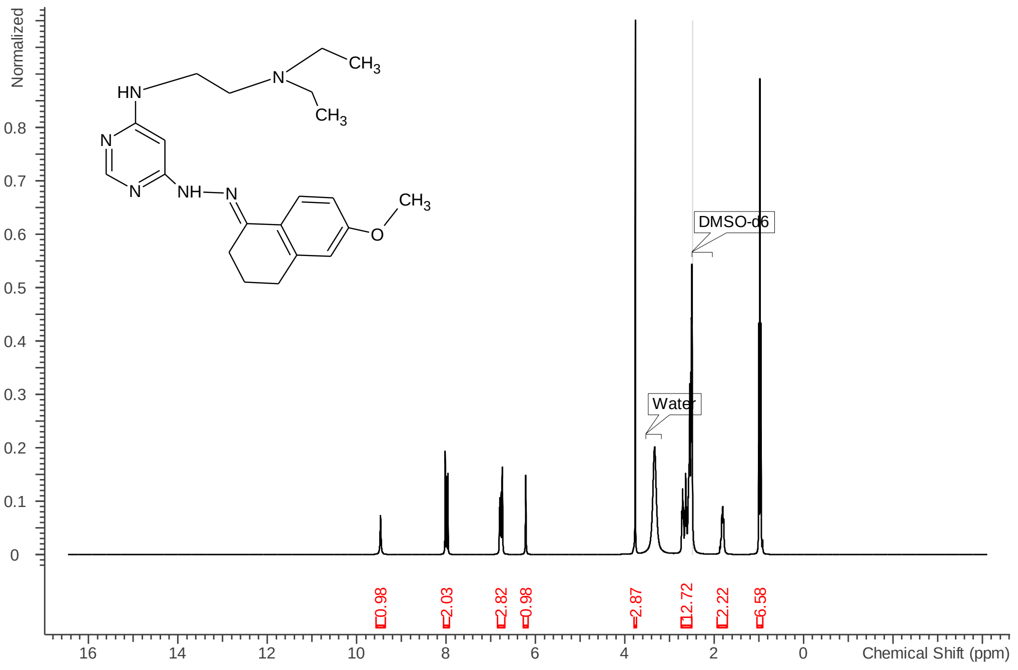

Figure S7. ^{1}H NMR spectrum of compound **4**.

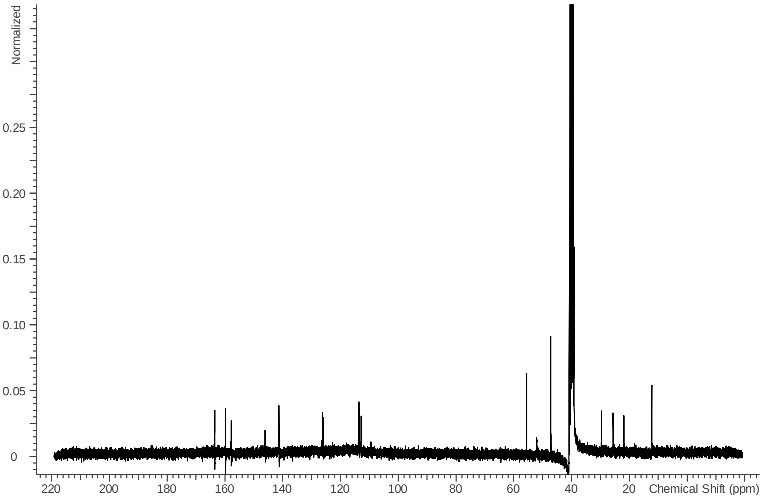

Figure S8. ^{13}C NMR spectrum of compound **4**.

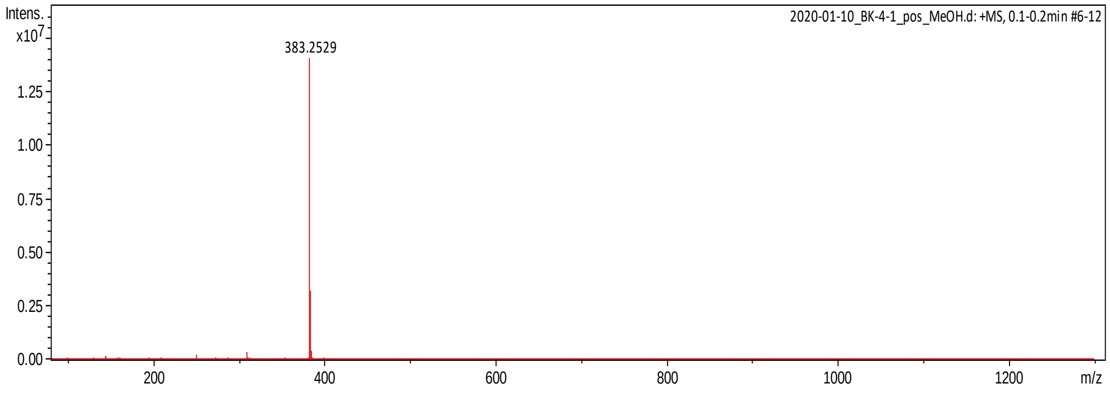

Figure S9. ESI-MS spectrum of compound **4.**

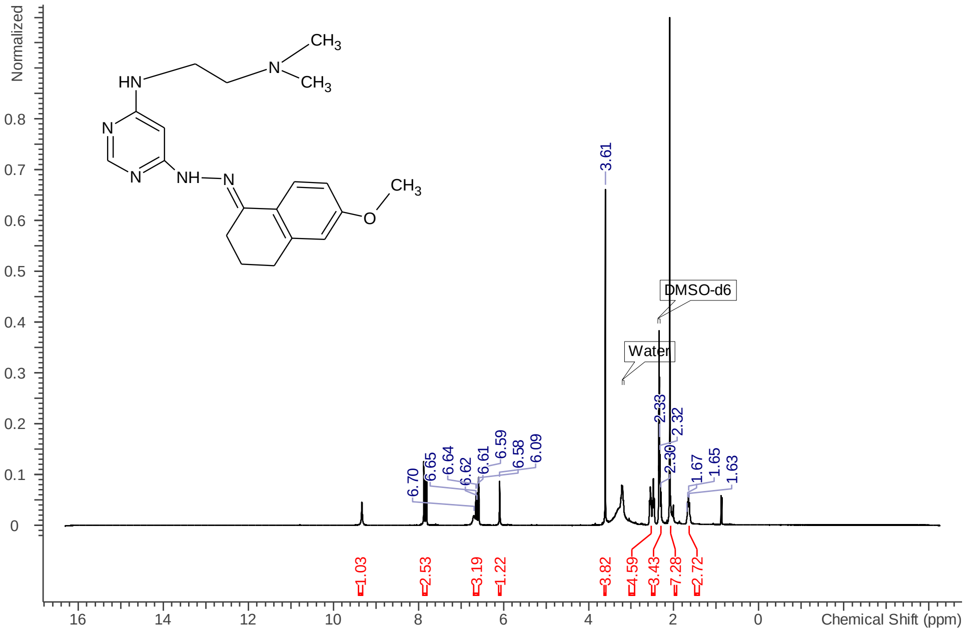

Figure S10. ^{1}H NMR spectrum of compound **5**.

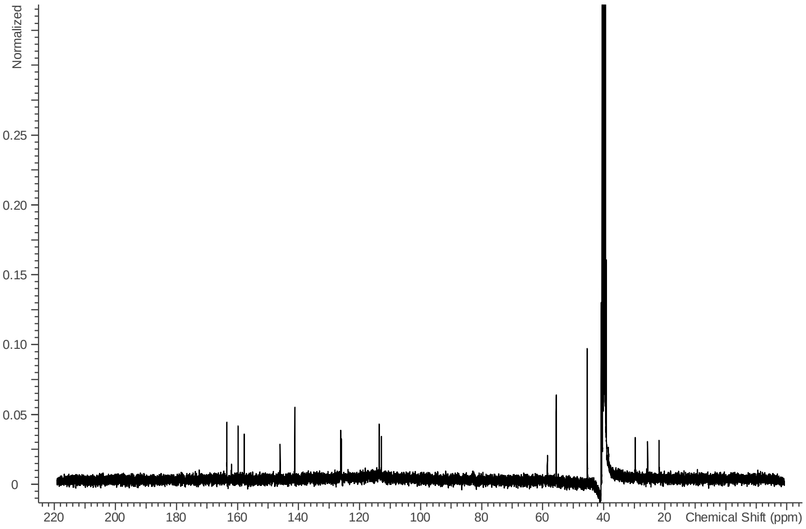

Figure S11. ^{13}C NMR spectrum of compound **5**.

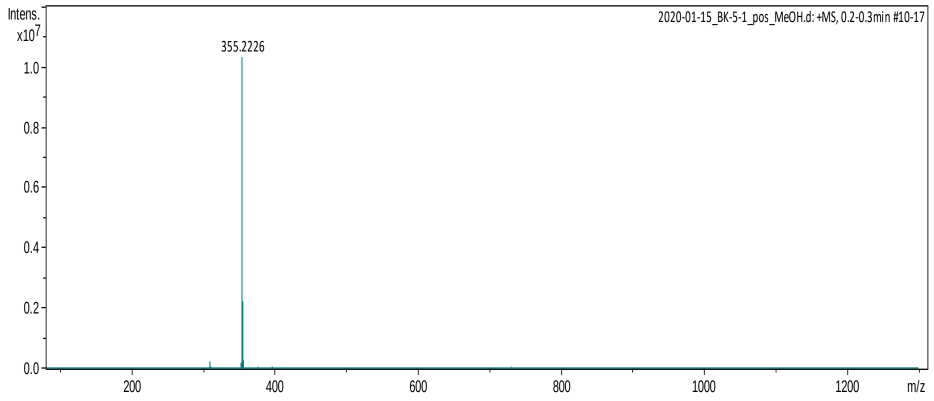

Figure S12. ESI-MS spectrum of compound **5**.

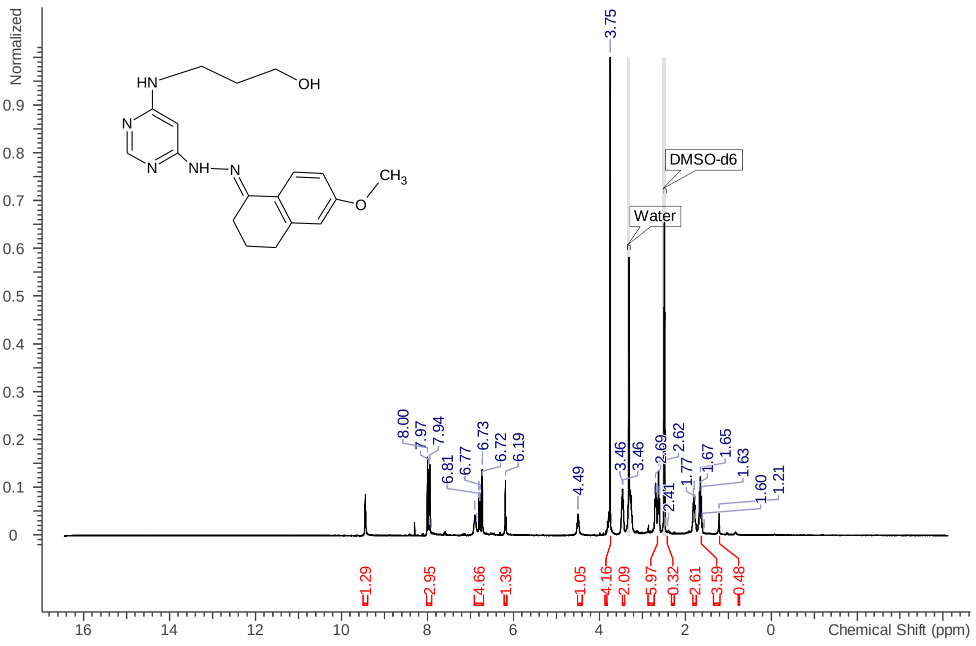

Figure S13. ^{1}H NMR spectrum of compound **6**.

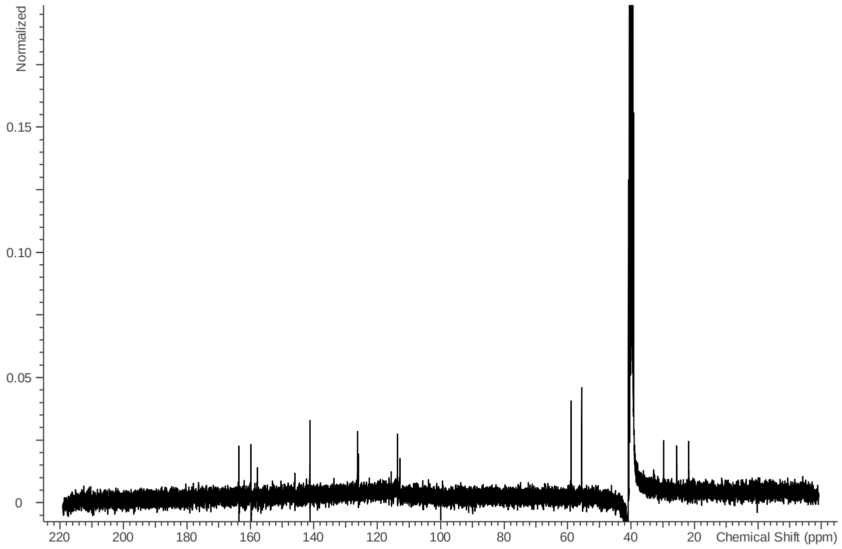

Figure S14. ^{13}C NMR spectrum of compound **6**.

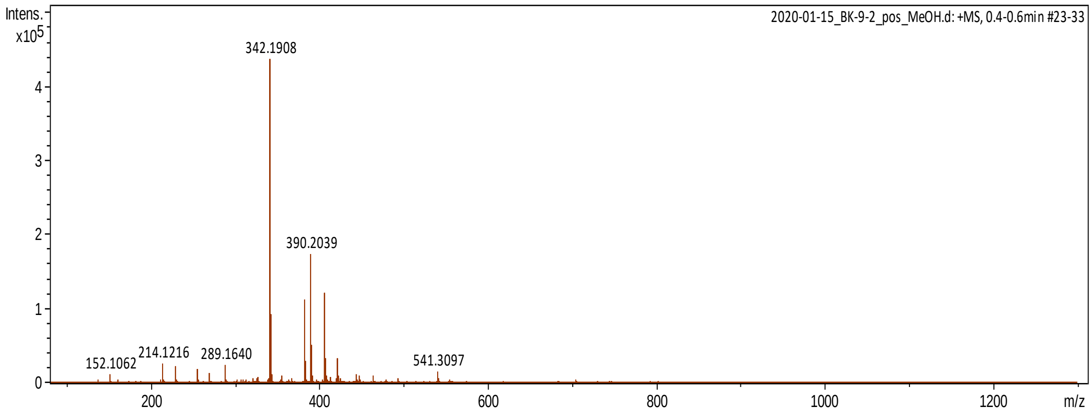

Figure S15. ESI-MS spectrum of compound **6**.

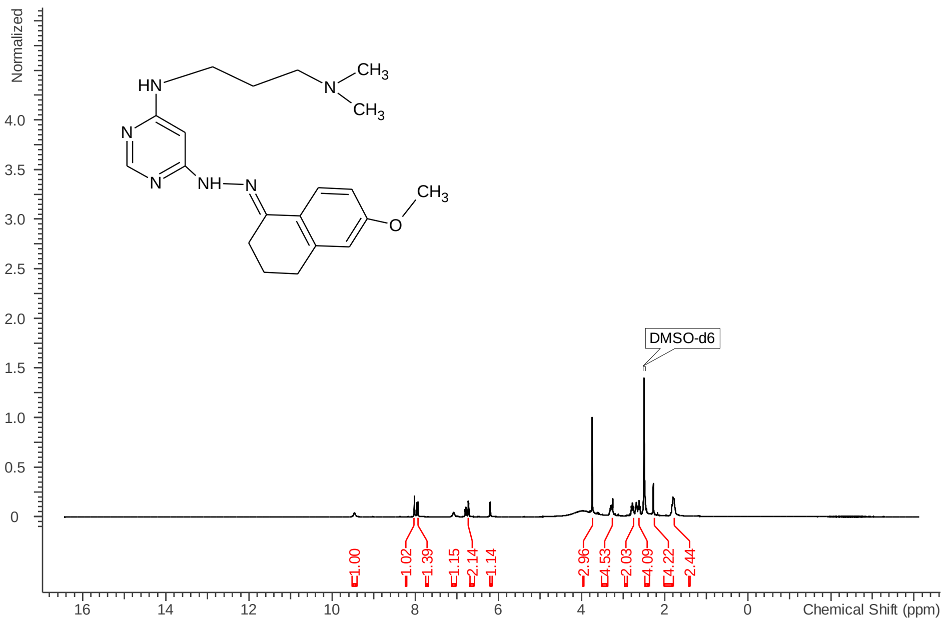

Figure S16. ^{1}H NMR spectrum of compound **7.**

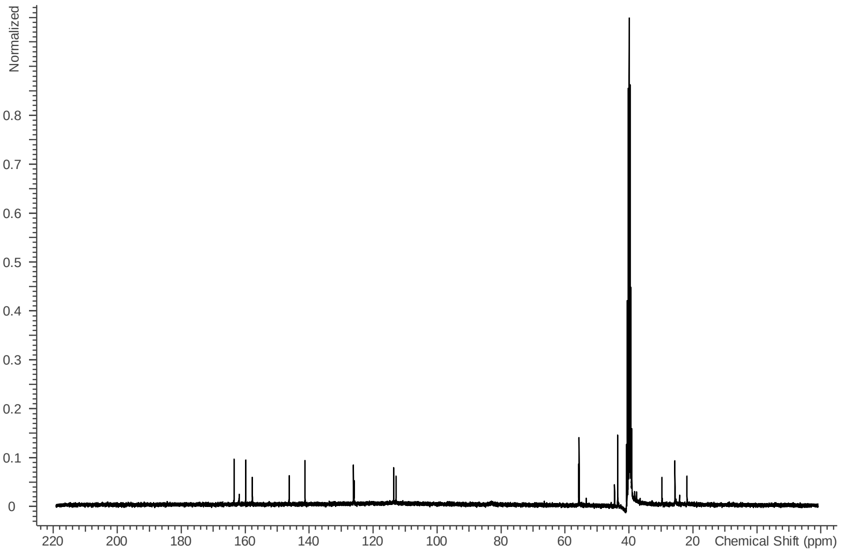

Figure S17. ^{13}C NMR spectrum of compound **7**.

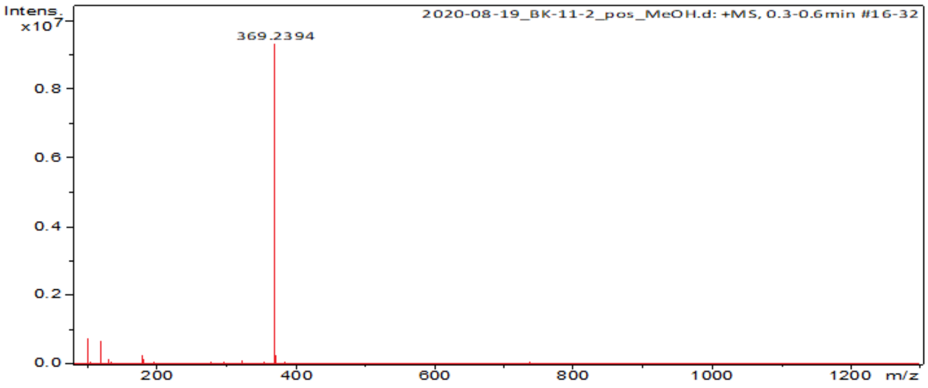

Figure S18. ESI-MS spectrum of compound **7**.
